# A simple and rapid approach to develop recombinant avian herpesvirus vectored vaccines using CRISPR/Cas9 system

**DOI:** 10.1016/j.vaccine.2017.12.025

**Published:** 2018-01-29

**Authors:** Na Tang, Yaoyao Zhang, Miriam Pedrera, Pengxiang Chang, Susan Baigent, Katy Moffat, Zhiqiang Shen, Venugopal Nair, Yongxiu Yao

**Affiliations:** a The Pirbright Institute & UK-China Centre of Excellence for Research on Avian Diseases, Pirbright, Ash Road, Guildford, Surrey GU24 0NF, United Kingdom; b Binzhou Animal Science and Veterinary Medicine Academy & UK-China Centre of Excellence for Research on Avian Diseases, Binzhou 256600, Shandong, PR China

**Keywords:** CRISPR/Cas9, HVT, Cre-loxP, Recombinant vaccine

## Abstract

Herpesvirus of turkeys (HVT) has been successfully used as live vaccine against Marek's disease (MD) worldwide for more than 40 years either alone or in combination with other serotypes. HVT is also widely used as a vector platform for generation of recombinant vaccines against a number of avian diseases such as infectious bursal disease (IBD), Newcastle disease (ND) and avian influenza (AI) using conventional recombination methods or recombineering tools on cloned viral genomes. In the present study, we describe the application of CRISPR/Cas9-based genome editing as a rapid and efficient method of generating HVT recombinants expressing VP2 protein of IBDV. This approach offers an efficient method to introduce other viral antigens into the HVT genome for rapid development of recombinant vaccines.

## Highlights

• Development of a rapid pipeline for generating recombinant HVT-based vaccines.
• NHEJ repair pathway makes targeted insertion of the foreign gene more efficient.
• Incorporation of RFP cassette enables the easy identification of recombinant virus.
• The recombinant virus has similar growth rate as parental virus with stable inserts.

## 1 Introduction

Marek’s disease (MD) is a lymphoproliferative disease of chickens affecting poultry health and welfare worldwide. MD has been controlled for more than four decades by the widespread use of live attenuated vaccines derived from homologous Marek’s disease virus (MDV) or antigenically-related avian herpesviruses. One such MD vaccine is based on Meleagrid herpesvirus 1, commonly known as herpesvirus of turkeys (HVT), due to its antigenic relatedness with the pathogenic MDV-1 strains [1]. HVT vaccine is the first generation of MD vaccine used in the early 1970s which has dramatically reduced losses from MD. Despite the introduction of new generations of MD vaccines to protect birds from increasingly virulent MDV strains, HVT-based vaccines are still being used widely [2], particularly in combination with other strains to exploit the synergistic protective effects [3]. HVT is also widely used as a vaccine vector for expression of heterologous antigens against a number of avian diseases such as Newcastle disease (ND), avian influenza (AI), infectious bursal disease (IBD), infectious laryngotracheitis (ILT), avian leukosis and Eimeria [2], [4], [5], [6], [7], [8]. Such recombinant HVT-vectored vaccines confer excellent and long-lasting simultaneous protective immunity against MD and the second disease caused by the virus whose genes are inserted in the vector. These recombinant HVT vaccines are generated either by conventional homologous recombination in virus-infected cells or through recombineering techniques on full-length genomes cloned in bacterial artificial chromosome (BAC) [9]. However, the generation of recombinant HVT using these methods is time-consuming requiring the construction of transfer vectors and several rounds of plaque purifications to obtain the recombinant vaccine candidate.

The CRISPR (clustered regularly interspaced short palindromic repeat)-Cas system is a natural microbial immune mechanism against invading viruses and other genetic elements [10], [11], [12]. The type II CRISPR-Cas system consisting of the RNA-guided Cas9 endonuclease (from Streptococcus pyogenes), a single guide RNA (sgRNA) and the trans-activating crRNA (tracrRNA), has been developed for genome editing in eukaryotic cells [13], [14]. The CRISPR/Cas9 system has been a huge success in efficient generation of genetically modified cells and animal models [15], [16], [17], [18], [19], [20]. It has also been used to manipulate genomes of several large DNA viruses, including herpes simplex virus type I, adenovirus, pseudorabies virus, vaccinia virus, Epstein-Barr virus, guinea pig cytomegalovirus and duck enteritis virus [21], [22], [23], [24], [25], [26], [27], [28], [29], [30]. Recently, we reported an efficient method using the CRISPR/Cas9 system to edit avian herpesvirus genomes [31].

In the present study, we developed a pipeline for rapid and efficient CRISPR/Cas9-mediated genome editing for generating recombinant HVT. Here we describe the use of this pipeline for generating an HVT-vectored vaccine harbouring VP2 gene of IBDV in the UL45/46 locus. The recombinant HVT vaccine candidate was evaluated in vitro for the expression and stability of inserted gene. The results from our studies demonstrate that CRISPR/Cas9-mediated gene editing is an alternative to traditional recombination and BAC recombineering techniques for the successful generation of recombinant HVT vaccine.

## 2 Materials and methods

### 2.1 Cell culture and virus

Primary chick embryo fibroblasts (CEF) were prepared from 10-day old embryos and maintained in M199 medium (Thermo Fisher Scientific) supplemented with 5% fetal bovine serum (FBS, Sigma), 100 units/ml of penicillin and streptomycin (Thermo Fisher Scientific), 0.25 µg/ml Fungizone (Sigma), and 10% tryptose phosphate broth (Sigma). HVT Fc126 strain, obtained from the Avian Disease and Oncology Laboratory (ADOL) East Lansing, MI, USA, was used for the construction of the recombinant candidate.

### 2.2 Construction of sgRNAs and donor plasmids

The gRNA targeting the UL45/46 region of the HVT genome was designed using CRISPR guide RNA designing software (http://crispr.mit.edu/) and cloned into the CRISPR/Cas9 vector pX459-v2 by introducing synthesized oligo-DNA primers corresponding to the target sequence into *Bbs*I restriction sites. The sg-A sequence was taken from published data [32] and cloned into px459-v2 in the same way. For construction of the donor plasmid containing the RFP expression cassette, the oligo pairs RFP-F and RFP-R (containing sg-A target sequence at both ends and a PacI site in the middle) were annealed and cloned into pGEM-T-easy vector. The RFP expression cassette was released by PacI restriction digestion from pEF-RFP and cloned into the resulting vector via the PacI site, generating donor plasmid pGEM-RFP. For construction of the donor plasmid containing RFP and VP2 expression cassettes, the oligo pairs RFP-VP2-F and RFP-VP2-R (containing the element of sgA + loxP+PacI + LoxP+SfiI + spacer + SfiI + sgA) were annealed and cloned into pGEM-T-easy vector generating pGEM-sgA-LoxP vector. The RFP expression cassette, released from pEF-RFP with restriction enzyme PacI, was cloned into pGEM-sgA-LoxP via the PacI site generating pGEM-sgA-LoxP-RFP. The VP2 expression cassette, which was PCR amplified from VAXXITEK®HVT-IBD with primer pairs VP2-F1 and VP2-R1, was then cloned into pGEM-sgA-LoxP-RFP via SfiI sites generating donor plasmid pGEM-sgA-LoxP-RFP-VP2. The primer sequences used for guide RNA cloning and donor plasmid construction are listed in Table 1.

**Table 1:** List of primers used for gRNA cloning, donor plasmid construction and recombinant virus characterization.

Primer	Sequence (5′–3′)
UL45/46-gRNA-F	CACCGAAAACACAGTAACCGTTAG
UL45/46-gRNA-R	AAACCTAACGGTTACTGTGTTTTC
sg-A-gRNA-F	CACCGAGATCGAGTGCCGCATCAC
sg-A-gRNA-R	AAACGTGATGCGGCACTCGATCTC
UL45/46-F	GATGCCCGCGTGTATCTTCA
UL45/46-R	ACGTAGGCTGAAAGTGTCCAG
UL45-F	TGTCGGCAGACTGTCCTGTA
VP2-R	GTGCATGACCGTGCTGATTC
VP2-F	GACCGGCGGCCGCCTAGGCCGGATCCCCCAACTCCGCCCGTTTTA
UL45/46-R	ACGTAGGCTGAAAGTGTCCAG
VP2-3F	CGTCTTGGCATCAAGACCGT
RFP-F	GAGATCGAGTGCCGCATCACCGGTTAATTAAGAGATCGAGTGCCGCATCACCGGA
RFP-R	CCGGTGATGCGGCACTCGATCTCTTAATTAACCGGTGATGCGGCACTCGATCTCA
VP2-F1	GACCGGCGGCCGCCTAGGCCGGATCCCCCAACTCCGCCCGTTTTA
VP2-R1	GACCGGCGGCCATAATGGCCGTCGACTCTAGAGGATCCGA
RFP-VP2-F	GAGATCGAGTGCCGCATCACCGGATAACTTCGTATAATGTATGCTATACGAAGTTATTTAATTAAATAACTTCGTATAATGTATGCTATACGAAGTTATGGCCGCCTAGGCCGGCGCGCCGTTTAAACGGCCATTATGGCCGAGATCGAGTGCCGCATCACCGGA
RFP-VP2-R	CCGGTGATGCGGCACTCGATCTCGGCCATAATGGCCGTTTAAACGGCGCGCCGGCCTAGGCGGCCATAACTTCGTATAGCATACATTATACGAAGTTATTTAATTAAATAACTTCGTATAGCATACATTATACGAAGTTATCCGGTGATGCGGCACTCGATCTCA

### 2.3 Generation of recombinant HVT candidates

Primary CEF were plated into 6-well plates the day before transfection. 0.5 µg of each gRNA were co-transfected with 1 µg donor plasmid into CEF cells using Lipofectamine® Reagent (Thermo Fisher Scientific) according to the manufacturer’s instructions. At 12 h post transfection, the CEF cells were infected with HVT at a multiplicity of infection of 0.01 plaque forming units (pfu)/cell. The infected CEF were passed three days later and the fluorescent marker was used for picking plaques to make virus stocks. For the excision of RFP using Cre recombinase, 2 µg of pcDNA3-Cre was transfected into CEF in 6-well plates. Twenty-four hours post transfection, the cells were infected with 100–200 pfu of HVT-RFP-VP2. Three days later, RFP-negative plaques were picked and used to infect CEF in 6-well plates, to purify further. The excision of RFP from the virus by Cre recombinase was confirmed by PCR of the RFP cassette using primers UL45-F and VP2-R. As a positive control, PCR of the VP2 cassette was used with primers VP2-F and UL45/46-R. The sequences of all the primers used in this study are listed in Table 1.

### 2.4 Characterization of recombinant HVT viruses

CEF cells were plated in six-well plates and then infected with purified HVT-RFP, or HVT-RFP-VP2, or HVT-VP2 the following day. The infected cells were harvested after 72 h of infection and lysed in 1 × squishing buffer (10 mM Tris-HCl, pH 8, 1 mM EDTA, 25 mM NaCl, and 200 µg/ml Proteinase K) at 65 °C for 30 min. PCR targeting the junction regions was carried out using primers UL45/46-F and UL45/46-R for identification of the recombinant HVT-RFP with the correct knock-in of the inserted gene. Similarly, Primer pair UL45-F and VP2-R (for RFP cassette) and primer pair VP2-F and UL45/46-R (for VP2 cassette) were used for identification of recombinant HVT-RFP-VP2 and HVT-VP2 viruses, respectively. As a positive control, PCR amplifying the VP2 cassette was carried out using primers VP2-F and UL45/46-R. The PCR products were purified using GenElute Gel Extraction Kit (Sigma) and sequence determined using primers listed in Table 1.

### 2.5 Western blot analysis

Expression of VP2 in recombinant virus-infected CEF cells was determined by western blot analysis using anti-VP2 monoclonal antibody (Mab) (Clone ID: EU0205 CAEU Company, Beijing, China, which specifically recognizes amino acids 394–410 of VP2) as the primary antibody. Mab against the HVT-encoded Bcl-2 homologue vNr-13 (clone EG8 generated at Pirbright Institute) was used as the HVT infection control. Briefly, 1 × 10^6^ infected cells were boiled with TruPAGE^TM^ LDS sample buffer (Sigma) for 10 min. The samples were separated on a 4–12% TruPAGE^TM^ Precast Gel, and the resolved proteins were transferred onto PVDF membranes. Immunoblots were blocked with 5% skimmed milk, and then incubated with anti-VP2 and anti-vNr-13 antibodies. After probing with primary antibodies, the blots were incubated with secondary antibody IRDye®680RD goat anti-mouse IgG (LI-COR) and visualized using Odyssey Clx (LI-COR).

### 2.6 Indirect immunofluorescence analysis (IFA)

The expression of VP2 in recombinant virus-infected cells was evaluated by immunofluorescence assays using confocal microscopy. CEF cells grown on coverslips in 24-well plates were infected with the recombinant viruses for 48–72 h before harvesting. After fixing with 4% paraformaldehyde and permeabilization with 0.1% Triton X-100, VP2 expression was analysed using anti-VP2 Mab (clone HH7 generated at Pirbright Institute). Chicken serum from HVT-vaccinated birds was used as the positive control for detecting HVT infection. Mixture of secondary antibodies containing goat anti-chicken IgG labelled with Alexa Fluor 488 and goat anti-mouse IgG labelled with Alexa Fluor 568 (Invitrogen) was used for the detection of specific fluorescence. Cell nuclei were then stained with 4′,6-diamidino-2-phenylindole (DAPI), and the images were taken using a Leica TCS SP5 confocal laser scanning microscope (Leica Microsystems).

### 2.7 Stability of the inserted genes in the recombinant viruses

The recombinant viruses were grown sequentially in CEF cells for 20 passages. Expression of VP2 was examined after every 5th passage by IFA and the integrity of the VP2 gene insert was examined by PCR (with primer pairs VP2-3F and UL45/46-R which amplifies the junction of VP2 cassette and UL46) using DNA extracted from every 5th passage.

### 2.8 In vitro growth kinetics

To investigate the growth properties of the recombinant HVT, CEF in 6-well plates were infected with 100 pfu of each virus and harvested at 0, 12, 24, 48, 72, 96 and 120 h post infection. DNA was extracted using the DNeasy 96 Blood & Tissue kit (Qiagen) and used for real-time q-PCR to determine the in vitro growth kinetics of the viruses using methods described previously [9]. Duplex real-time q-PCR to detect both HVT SORF1 gene and chicken ovotransferrin gene enabled calculation of HVT genome copies per 10, 000 cells using dilution series of pHVT BAC3 DNA [9] and p-GEM-T-ovo [33] to produce standard curves. The HVT genome copies per 10,000 cells was plotted against hours post-infection for each of the viruses.

## 3 Results

### 3.1 Targeted knock-in of RFP expression cassette into the HVT genome using CRISPR/Cas9 system

**Fig. 1:**
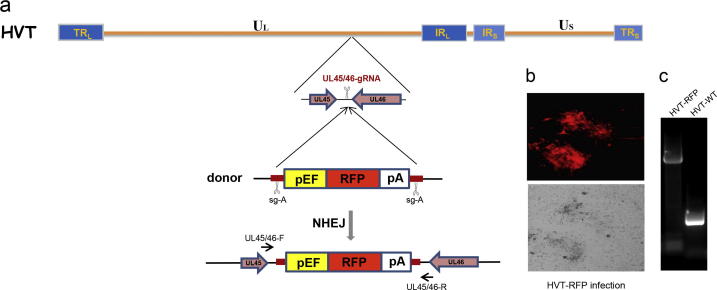
Homology-independent knock-in of reporter gene into CRISPR/Cas9-induced DSBs in HVT genome. (a) Schematics of the donor plasmid and targeting strategy for CRISPR/Cas9-induced homology-independent insertion of RFP reporter at UL45 and UL46 gene junction. The internal and terminal repeat long (TR_L_/IR_L_) and short (TR_S_/IR_S_) regions flanking the unique long (U_L_) and short (U_S_) regions of the HVT genome are shown. Genomic positions and orientations of UL45 and UL46 are shown. The RFP expression cassette is flanked with sg-A target sites. (b) CEF were infected with recombinant HVT-RFP virus and visualized by immunofluorescence and bright field. (c) CEF infected with wild-type (WT) or the isolated HVT-RFP mutant were collected for PCR analysis using the primers as shown in (a).

To exclude any potential adverse effects of gene modification on viral replication, the UL45/UL46 intergenic junction, previously proven to be suitable for insertion of foreign genes in several studies [5], [7], [34], was selected as the target locus for the generation of recombinant HVT in our study (Fig. 1). In order to determine whether the CRISPR-Cas9 system could be used to knock-in a foreign gene at the above target locus, we initially attempted to knock-in the RFP expression cassette. Considering the higher efficiency of the non-homologous end-joining (NHEJ) pathway over the homology-directed repair (HDR) pathway [32], [35], [36], [37], together with the evidence that the linearized foreign gene was efficiently captured at a DNA double strand break (DSB) in the genomes of mammalian cells, zebrafish and Pseudorabies virus through a homology-independent DNA repair mechanism [24], [32], [38], [39], we used the NHEJ pathway for knocking-in the foreign gene expression cassette into HVT genome.

We constructed a donor plasmid that carries the RFP reporter gene cassette flanked by the sgRNA (sg-A) target sites [32] to introduce cleavage for the generation of RFP expression fragment for designed integration. Two gRNAs, sg-A for targeting the donor plasmid to release the insert fragment and UL45/46-gRNA for targeting viral genome at UL45/46 gene junction, were cloned into pX459-v2 (Addgene plasmid # 62988) that expresses the codon-optimized S. pyogenes Cas9 (Cas9) as a bicistronic mRNA with the puromycin-N-acetyltransferase gene and the gRNA sequence driven by the U6 promoter. The concurrent cleavage of donor plasmid DNA and the targeted viral genome by Cas9 will introduce the RFP cassette into UL45/UL46 locus as shown in Fig. 1a. We co-transfected the donor plasmid together with Cas9/sgRNA vectors targeting both donor plasmid and gene junction UL45/UL46 into CEF. Eight hours post transfection, the cells were then infected with wild type (WT) HVT. The transfected/infected cells were passed three days post infection (dpi) and incubated until red fluorescent plaques were observed. The progeny virus plaques carrying the knock-in cassette of RFP were isolated and designated HVT-RFP (Fig. 1b). The presence of RFP cassette at the UL45/UL46 locus was confirmed by PCR amplification with site-specific primers (Fig. 1c). Subsequent analysis of the junction sequences revealed indel events, indicating that the error-prone NHEJ-mediated DNA repair mechanism has taken place (data not shown).

### 3.2 Targeted knock-in of VP2 expression cassette into the HVT genome

**Fig. 2:**
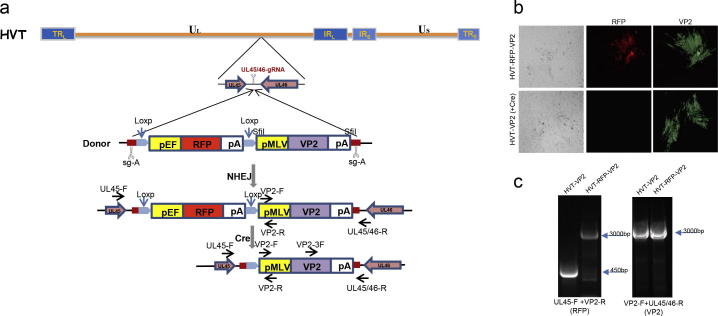
Homology-independent knock-in of VP2 in HVT genome. (a) Schematics of the donor plasmid and targeting strategy for RFP-VP2 knock-in at UL45 and UL46 gene junction and RFP excision by Cre recombinase. The RFP expression cassette is flanked with LoxP sites and followed by VP2 expression cassette. The two expression cassettes are flanked by sg-A target sites. (b) CEF were infected with recombinant HVT-RFP-VP2 (top panel) and HVT-VP2 (bottom panel), stained with anti-VP2 monoclonal antibody HH7 and visualized by immunofluorescence and bright field. (c) CEF infected with HVT-RFP-VP2 and HVT-VP2 viruses were collected for PCR analysis using the primers as shown in (a).

Having demonstrated that the RFP expression cassette could be efficiently inserted into the targeted site by CRISPR/Cas9 system, we examined the insertion of IBDV VP2 cassette for the construction of recombinant vaccine against IBDV. To establish an efficient and rapid strategy for the development of recombinant vaccines, we combined CRISPR/Cas9 and Cre/Lox systems where the IBDV VP2 expression cassette was placed next to an RFP expression cassette flanked with LoxP sites [40] to facilitate marker gene excision by Cre recombinase (Fig. 2a). The two cassettes are flanked by the sg-A target sites to generate insert for designed integration into the HVT UL45/46 loci. After co-transfection of the donor plasmid together with Cas9/sgRNA vectors and infection with HVT, recombinant HVT-RFP-VP2 plaques expressing RFP were isolated.

The RFP expression cassette was excised from HVT-RFP-VP2 virus with Cre recombinase using the expression plasmid pcDNA3-Cre transfected into CEF 24 h before infection with HVT-RFP-VP2. The RFP-negative plaques from which the RFP expression cassette was successfully excised were further purified in CEF to obtain recombinant virus stocks (Fig. 2b). Deletion of RFP from the recombinant virus stocks was confirmed by PCR using primer pairs specific for RFP and VP2 cassettes. As expected, the RFP expression cassette was deleted from the virus stocks while the VP2 expression cassette remained intact (Fig. 2c). The resulting mutant HVT was designated HVT-VP2.

### 3.3 Expression of VP2 in HVT-VP2 infected CEF

**Fig. 3:**
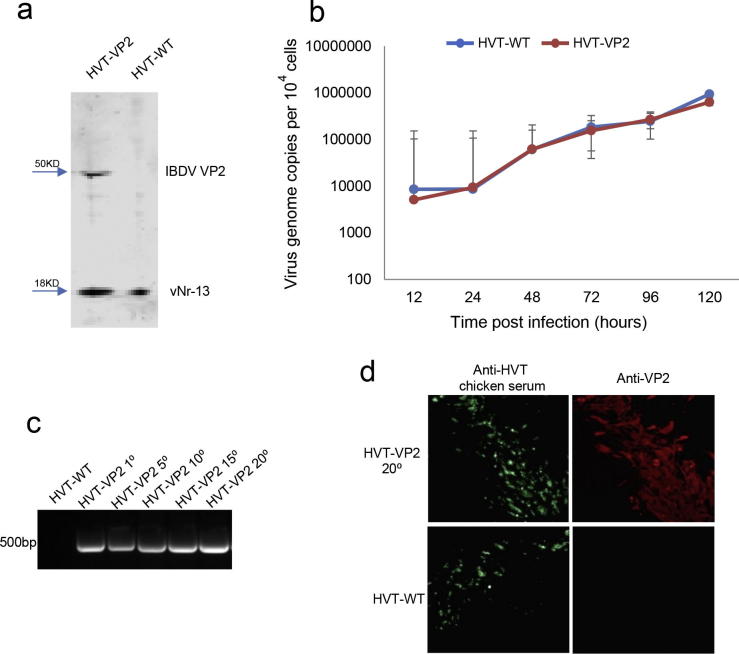
Characterization of the recombinant HVT-VP2. (a) Detection of VP2 expression by western blotting. HVT-encoded Bcl-2 homolog vNr-13 was detected as confirmation of HVT infection. (b) The growth kinetics of recombinant HVT-VP2. In vitro growth rates of HVT-WT and HVT-VP2 measured from the viral genome copy numbers determined using TaqMan real-time qPCR on DNA extracted from CEF harvested at various time points after inoculation. Viral genome copy numbers per 10,000 cells (shown with 95% confidence intervals) are shown on the y axis. (c) Junction PCR to confirm the presence of VP2 expression cassette from the recombinant viruses at passage 1, 5, 10, 15 and 20 in CEFs using primers shown in Fig. 2a. (d) Detection of VP2 expression from the recombinant viruses passaged 20 times in CEFs with IFA using anti-VP2 monoclonal antibody HH7. HVT infection is confirmed by immunostaining with HVT infected chicken serum.

We next investigated whether the inserted VP2 gene could be expressed in recombinant HVT-VP2 infected CEF. Expression of VP2 protein was assessed by western blotting using lysates of cells infected with WT HVT or recombinant HVT-VP2 using IBDV VP2-specific mouse Mab EU0205. HVT-vNr-13-specific Mab EG8 was used as an infection/loading control. As expected, the cell lysates from HVT-VP2 infected cells demonstrated a 50 kDa-sized VP2 protein (Fig. 3a), that was clearly absent in the cell lysates infected with parental HVT.

### 3.4 In vitro replication and stability of recombinant HVT-VP2

Having confirmed the expression of VP2 in recombinant HVT-VP2, we determined whether the insertion of the VP2 expression cassette did influence the growth of the recombinant HVT-VP2 compared to the parental HVT. For this, primary CEFs in 6-well plate were infected with 100 pfu of either HVT or recombinant HVT-VP2. Virus replication rates measured by qPCR that determines the viral genome copy numbers per 10,000 cells at various dpi did not show significant differences between the recombinant and the parental viruses (Fig. 3b).

To determine the stability of the expression cassette during continuous passage, recombinant virus was grown on primary CEFs sequentially for 20 passages and viral DNA was extracted and analysed after every 5 passages using VP2-specific PCR. There was no variability in the size and amount of VP2 gene product following repeated passages of the virus in cultured cells, indicating that the VP2 gene was stably inserted into the HVT genome (Fig. 3c). Similarly, the expression of VP2 was observed when HVT-VP2 infected cells were analysed after every 5 passages by IFA (Fig. 3d, result of 20th passage). Sequential passage showed no effect on the replication ability of the recombinant virus. These data suggest that VP2 gene was stably integrated in the HVT genome exerting no adverse effects on replication.

## 4 Discussion

Vaccines used currently against MD include attenuated serotype 1 strain (CVI988/Rispens), avirulent serotype 2 (SB-1), and serotype 3 HVT, with advantages of overcoming the inhibitory effects of maternal antibodies and inducing long-term protective immunity in chickens. All of these vaccine strains also have the potential for use as recombinant vaccine vectors due to their ability to take insertions of foreign genes. However, only HVT vector has been commercially used as a vaccine vector for expression of heterologous antigens as it is highly safe for chickens and grows remarkably well in cell culture. While conventional recombination strategies or recombineering methods with cosmid or BAC clones of the HVT were widely used for the generation of recombinant vaccines, the advent of the revolutionary gene editing technologies, mainly using the CRISPR/Cas9 nucleases, has opened up the possibilities of using them for editing the genomes of these viruses. We have demonstrated the feasibility of the technique by generating recombinant HVT with mutations in a number of essential and non-essential genes [31]. The present study is to advance the application of the CRISPR/Cas9-based gene editing tools as a pipeline to develop HVT-based novel recombinant vaccines to protect against major avian diseases.

To our knowledge, this is the first study to demonstrate effective use of the CRISPR/Cas9 system in developing the recombinant HVT vaccine. Compared with traditional technologies for modification of HVT, such as homologous recombination [7] and BAC mutagenesis technology [5], the novel CRISPR/Cas9 approach is more convenient and efficient. The double stranded breaks (DSBs) created by Cas9 are repaired by one of the two general repair pathways: NHEJ and HDR pathways [41], [42]. NHEJ is more efficient than HDR as NHEJ occurs throughout the cell cycle [43], whereas HDR only occurs during S and G2 phases [16], [44], [45]. We took advantage of higher efficiency of NHEJ repair pathway to introduce the foreign genes into the targeted locations. The use of universal gRNA target site flanking the foreign gene cassette could speed up the procedure dramatically as the donor template could be constructed straight away without the need waiting for the specific gRNA selection. As a proof of principle, we first demonstrated the efficient knock-in of a RFP expression cassette. Next we created the repair donor vector which has the features of an excisable RFP selection marker flanked by LoxP sites and unique restriction enzyme sites SfiI for swapping the foreign gene expression cassette of interest. Combination of this donor vector along with gRNA-guided Cas9 construct was used for the efficient and rapid production of recombinant HVT vaccine expressing the heterologous antigen, IBDV VP2 in this case. After generation of the recombinant HVT-RFP-VP2 virus using the donor vector containing RFP and VP2 expression cassettes, RFP was successfully removed by Cre recombinase expression. We also showed that the insertion of VP2 expression cassette did not have any effect on HVT replication, and that the inserted cassette remained stable even after 20 passages as confirmed by both PCR and immunostaining. Thus the combination of the CRISPR/Cas9 and Cre-LoxP systems allows rapid development of recombinant HVT-based vector vaccines.

In summary, we have demonstrated here that the CRISPR/Cas9 system is a versatile and powerful technology for the development of innovative HVT vectored vaccines. Although the insertion of only a single foreign antigen VP2 was tested here, it will be feasible to try the same approach to develop multivalent recombinant HVT vectored vaccines by inserting other viral antigen genes in more genomic locations of the virus. In addition to the UL45/46 loci used here, the HVT65/66 locus used for VP2 insertion in Vaxxitek HVT-IBD (Boehringer Ingelheim) and the US2 locus used for insertion of the bacterial miniF plasmid in the pHVT BAC [9] are already known as suitable loci for insertion of foreign genes. The same approach can also be extended to engineer SB-1 and CVI988 genomes for the development of new multivalent vectored vaccines for protecting multiple poultry diseases. There are also the prospects of applying the technology to other avian DNA viruses including adenoviruses, pox viruses and other herpesvirus such as infectious laryngotracheitis virus. Application of this CRISPR/Cas9 system platform to develop new multivalent vectored vaccines will be highly beneficial for protecting against multiple poultry diseases.

## Conflict of interest

The authors report no conflict of interest.

## Funding information

This project was supported by the Biotechnology and Biological Sciences Research Council (BBSRC) grants BB/P016472/1 and BB/L014262/1, and the Royal Society International Collaboration Award for Research Professors (Ref IC 160046).
